# EEG Signal Reconstruction Using a Generative Adversarial Network With Wasserstein Distance and Temporal-Spatial-Frequency Loss

**DOI:** 10.3389/fninf.2020.00015

**Published:** 2020-04-30

**Authors:** Tian-jian Luo, Yachao Fan, Lifei Chen, Gongde Guo, Changle Zhou

**Affiliations:** ^1^College of Mathematics and Informatics, Fujian Normal University, Fuzhou, China; ^2^School of Informatics, Xiamen University, Xiamen, China; ^3^Digital Fujian Internet-of-Thing Laboratory of Environmental Monitoring, Fujian Normal University, Fuzhou, China

**Keywords:** EEG signals reconstruction, generative adversarial network, Wasserstein distance, sampling rate, sensitivity

## Abstract

Applications based on electroencephalography (EEG) signals suffer from the mutual contradiction of high classification performance vs. low cost. The nature of this contradiction makes EEG signal reconstruction with high sampling rates and sensitivity challenging. Conventional reconstruction algorithms lead to loss of the representative details of brain activity and suffer from remaining artifacts because such algorithms only aim to minimize the temporal mean-squared-error (MSE) under generic penalties. Instead of using temporal MSE according to conventional mathematical models, this paper introduces a novel reconstruction algorithm based on generative adversarial networks with the Wasserstein distance (WGAN) and a temporal-spatial-frequency (TSF-MSE) loss function. The carefully designed TSF-MSE-based loss function reconstructs signals by computing the MSE from time-series features, common spatial pattern features, and power spectral density features. Promising reconstruction and classification results are obtained from three motor-related EEG signal datasets with different sampling rates and sensitivities. Our proposed method significantly improves classification performances of EEG signals reconstructions with the same sensitivity and the average classification accuracy improvements of EEG signals reconstruction with different sensitivities. By introducing the WGAN reconstruction model with TSF-MSE loss function, the proposed method is beneficial for the requirements of high classification performance and low cost and is convenient for the design of high-performance brain computer interface systems.

## 1. Introduction

Electroencephalography (EEG) (Cecotti and Graser, 2011; Narizzano et al., 2017; Freche et al., 2018) is one of the most important non-invasive neuroimaging modalities used in cognitive neuroscience research (Mullen et al., 2015; Mete et al., 2016; Luo et al., 2018b) and brain-computer interface (BCI) development (Ahn and Jun, 2015; Arnulfo et al., 2015; Sargolzaei et al., 2015; Kumar et al., 2017). However, EEG-based cognitive neuroscience and BCI fields currently face a bottleneck in that high sampling rate and high-sensitivity EEG amplifier hardware are extremely expensive and generally complicated to operate for collecting signals (Jiang et al., 2017). Ideally, EEG amplifiers with high sampling rates and sensitivities are preferred to record high-resolution brain activities underlying different stimuli. Lowering the sampling rate and sensitivity may influence the utility of acquired signals (Wu et al., 2015). Therefore, extensive efforts have been dedicated to reconstructing high-sampling-sensitivity EEG (HSS-EEG) signals from low-sampling-sensitivity EEG (LSS-EEG) signals to improve performance. The up-sampling operation is one of the conventional time-series reconstruction methods. By using an up-sampling operation, the reconstructed signals are up-sampled and with different sensitivity. The reconstruction methods can be divided into three categories:

1. Reconstruction by interpolation (Erkorkmaz, 2015).
2. Reconstruction by mathematical modeling (Naldi et al., 2017).
3. Reconstruction by deep neural networks (Jin et al., 2017).

Among the methods for reconstructing EEG signals by interpolation algorithms, such as bilinear interpolation, nearest neighbor interpolation, and spline interpolation, several are based on the successive assumption of signal values (Marques et al., 2016). Such an assumption does not consider the complexity of signals, and, therefore, it is difficult to represent brain activity from reconstructed signals. Reconstruction based on mathematical models, such as compressive sensing, subspace projection, and frequency transformation, optimizes an objective function that incorporates mathematical models and prior information in the different domains of the signals. These algorithms greatly improve signal performance and quality; however, they may still lose the details representing brain activity and suffer from artifacts. In addition, reconstruction by a single mathematical model and a single domain has simplified the range of applications of reconstructed EEG signals. These algorithms greatly improve signal performance and quality (Choudhary et al., 2016); however, they may still lose the details representing brain activity and suffer from artifacts. Additionally, the high computational cost of constructing mathematical models remains another potential risk in practical applications.

In contrast to interpolation and mathematical models, the recent explosive development of deep neural networks (DNNs) has shed light on novel opinions and promised potential in the field of signal reconstruction. In recent years, most DNNs studies have focused on image signal reconstruction from the perspective of noise, super-resolution, and denoising (LeCun et al., 2015). A state-of-the-art image reconstruction performance was obtained by the new game theoretic generative model of generative adversarial networks (GANs) (Goodfellow et al., 2014). GANs are used to generate images from artificial data, construct high-resolution (HR) images from low-resolution (LR) copies (Ledig et al., 2017), and denoise CT images from noisy images (Yang et al., 2018), and such models achieve the best performance in reconstruction tasks. Inspired by the applications of GANs in the image reconstruction field, researchers have focused on reconstructing EEG signals using GANs. Research on “GANs conditioned by brain signals” (Kavasidis et al., 2017) has used GANs to generate images seen by subjects from recorded EEG signals. Another deep EEG super-resolution study used GANs to produce HR EEG data from LR samples by generating channel-wise up-sampled data to effectively interpolate numerous missing channels (Hartmann et al., 2018). Such an algorithm produced higher spatial resolution EEG signals to improve performance.

Although GANs have been used to reconstruct images from EEG signals with a visualized spatial feature space, the sampling rate and sensitivity resolution in the temporal feature space are still two key limitations of EEG signals. To counterbalance the performance of EEG signals and the cost of EEG amplifiers, we propose using a GAN with the Wasserstein distance (WGAN) model as the discrepancy measure between different sampling rates and sensitivities and a spatial-temporal-frequency loss function that computes the difference between EEG signals in an established feature space. The GAN/WGAN architecture is used to encourage the reconstructed LSS-EEG signals to share the same distribution as the HSS-EEG signals. Because EEG signals are multi-channels time-series data, instead of using the mean square error by temporal features as the loss function, we propose a novel spatial-temporal-frequency loss function, which is robust enough for the EEG signals, to extract the spatial-temporal-frequency features for reconstruction. By using the GAN/WGAN architecture and the carefully designed loss function to reconstruct HSS-EEG signals from LSS-EEG signals, this study has made two contributions:

1. The GAN/WGAN architectures are trained by EEG signals of different sampling rates and different sensitivities to compare the classification performances of the reconstructed EEG signals.
2. The spatial-temporal-frequency loss is applied to maintain robustness of GAN/WGAN architectures training, and the loss function helps reconstruction signals to obtain more discriminant patterns.

## 2. Methods

### 2.1. EEG Signal Reconstruction Model

For the reconstruction of EEG signals, let $z\in R^{N\times T_{1}\times S}$ denote the LSS-EEG signals from distribution *P*_*L*_, and $x\in R^{N\times T_{2}\times S}$ denote the HSS-EEG signals from the real distribution *P*_*H*_. In the definition, *N* denotes the number of channels, and *T*_1_ and *T*_2_ denote the samples of one trial for LSS-EEG signals and HSS-EEG signals during recordings, respectively. *S* denotes the number of trials for the motor-based tasks. The reconstruction goal is to formulate a function *f*(*z*) that projects LSS-EEG signals *z* to HSS-EEG signals *x*:

(1) $$\begin{array}{r} f\left(z\right):z\to x \end{array}$$

In fact, the reconstruction function maps the LSS-EEG samples from *P*_*L*_ into a certain distribution *P*_*C*_, and our goal is to adjust a certain distribution *P*_*C*_ to make it close to the real distribution *P*_*H*_ by varying the function *f*(*z*). The reconstruction has two procedures with GAN. In the generation procedure, the object is to adjust EEG samples from distribution *P*_*L*_ to distribution *P*_*C*_. In the discriminator procedure, the object is to adjust EEG samples from distribution *P*_*C*_ to distribution *P*_*H*_. The reconstruction procedure can ultimately be treated as a procedure to adjust EEG samples from one distribution to another.

Typically, since EEG signals are nonlinear and non-stationary, the noise model in such signals is complicated, and the reconstruction mapping relationship is non-uniformly distributed across the signals. Thus, there is no clear indication of how the distributions of LSS-EEG and HSS-EEG signals are related to each other. It is difficult to reconstruct LSS-EEG signals using conventional methods. However, the uncertainties in the noise model and the reconstruction mapping relationship can be ignored by using deep neural networks (DNNs), as the DNNs can efficiently learn high-level features from nonlinear and non-stationary signals and reconstruct a representation of the data distribution from modest-sized signal patches. Therefore, the GAN framework based on DNN is suitable for EEG signal reconstruction. In summary, a modified GAN framework with the Wasserstein distance and temporal-spatial-frequency (TSF) loss is introduced to reconstruct HSS-EEG signals from LSS-EEG signals.

### 2.2. GAN With Wasserstein Distance

The GAN framework consists of two opposing neural networks, a generator *G*, and a discriminator *D*, that are optimized to minimize a two-player min-max problem (Goodfellow et al., 2014). The discriminator is trained to distinguish the generated samples from the real samples, while the generator is trained to generate fake samples that are not determined as fake by the discriminator. For the reconstruction of EEG signals, we further defined the discriminator *D*_θ_*D*__ and the generator *G*_θ_*G*__ to solve the min-max problem:

(2) $$\begin{array}{rc} \begin{array}{c} min\\ \theta_{G} \end{array}\begin{array}{c} max\\ \theta_{D} \end{array}L_{GAN}\left(D_{\theta_{D}},G_{\theta_{G}}\right) & =E_{x\sim P_{H}}\left[logD_{\theta_{D}}\left(x\right)\right]\\ +E_{z\sim P_{L}}\left[log\left(1-D_{\theta_{D}}\left(G_{\theta_{G}}\left(z\right)\right)\right)\right] \end{array}$$

where *E*(·) denotes the expectation operator. When the discriminator meets the real data, it will satisfy *D*_θ_*D*__(*x*) = 1 to discriminate the real data. Here, *D*_θ_*D*__(*x*) = 1 reaches the expectation for *logD*_θ_*D*__(*x*). When the discriminator meets the generated data, it will satisfy *D*_θ_*D*__(*G*_θ_*G*__(*z*)) = 0 to discriminate the generated data. Here, *D*_θ_*D*__(*G*_θ_*G*__(*z*)) = 0 reaches the expectation for *log*(1−*D*_θ_*D*__(*G*_θ_*G*__(*z*))). Therefore, the minimax optimal function is designed by the expectation operator. The general reconstruction idea is to train a generator for the purpose of fooling a differentiable discriminator that is trained to distinguish reconstructed HSS-EEG signals from real HSS-EEG signals. In constructing EEG signals, GANs suffer from remarkable training difficulty due to the nonlinear and non-stationary characteristics of EEG signals. To overcome the training problem of the original GAN framework, instead of using Jensen–Shannon divergence, the WGAN framework uses the Wasserstein distance to compare sample distributions (Gulrajani et al., 2017). From the definition of WGAN, the min-max problem optimized by *D*_θ_*D*__ and *G*_θ_*G*__ can be written:

(3) $$\begin{array}{rc} \begin{array}{c} min\\ \theta_{G} \end{array}\begin{array}{c} max\\ \theta_{D} \end{array}L_{WGAN}\left(D_{\theta_{D}},G_{\theta_{G}}\right) & =-E_{x\sim P_{H}}\left[D_{\theta_{D}}\left(x\right)\right]\\ +E_{z\sim P_{L}}\left[D_{\theta_{D}}\left(G_{\theta_{G}}\left(z\right)\right)\right]\\ +\lambda E_{\tilde{x}\sim P_{R}}\left[{\left({\left||\nabla_{\tilde{x}}\left(D\left(\tilde{x}\right)\right)|\right|}_{2}-1\right)}^{2}\right] \end{array}$$

In the min–max problem, the Wasserstein distance is estimated by the first two terms. The last term is the gradient penalty for network regularization. In the penalty term, *P*_*R*_ denotes the distribution of uniform samples $\tilde{x}$ along straight lines connecting pairs of generated and real samples. $\nabla_{\tilde{x}}\left(\cdot \right)$ is the gradient calculator, and the parameter λ is a constant weighting parameter for the penalty term. In fact, the WGAN framework removes the log function and drops the last sigmoid layer to keep the gradient while training the min-max problem. The discriminator *D*_θ_*D*__ and the generator *G*_θ_*G*__ are trained alternatively by optimizing one and updating the other.

### 2.3. TSF-MSE Loss Function

To allow the generator to transform the data distribution from a low sampling rate and sensitivity to a high sampling rate and sensitivity, another part of the loss function needs to be added to the GAN/WGAN architecture to retain the detail and information content of the EEG signals. A widely used loss function for signal details and information contents is the mean square error (MSE) loss function (Yang et al., 2018). Typically, as the common MSE is computed by minimizing the point-wise error in image processing, the temporal MSE is computed by minimizing the time sampling point-wise error between a LSS-EEG patch and a HSS-EEG patch by the time step:

(4) $$\begin{array}{r} L_{T-MSE}\left(G_{\theta_{G}}\right)=E_{\left(x,z\right)}\left[\frac{1}{T^{2}}||G\left(z\left(t\right)\right)-x\left(t\right)|{|}_{F}^{2}\right] \end{array}$$

where ||·||_*F*_ denotes the Frobenius norm, *L*_*T*−*MSE*_ denotes the temporal MSE for the generator *G*_θ_*G*__, *t* is the time step of real EEG signals and generated EEG signals, and *T* is the number of time steps for each batch. In contrast to images, EEG signals are multi-channel time-series data, and the spatial and frequency features must be considered for reconstruction. Therefore, in addition to the temporal MSE *L*_*T*−*MSE*_ between time steps, the spatial MSE *L*_*S*−*MSE*_ between channels and the frequency MSE *L*_*F*−*MSE*_ between signal batches also need to be considered for encouraging the GAN/WGAN architecture to construct more accurate HSS-EEG signals. Recently, common spatial patterns (CSP) have been widely used to extract spatial features from EEG signals (Luo et al., 2018a), and power spectral density (PSD) features are widely used to extract frequency features from EEG signals (Petroff et al., 2016). The CSP algorithm is used to compute the optimal projection vectors to project the original EEG signal to a new space to obtain good spatial resolution and discrimination between different classes of EEG signals. The PSD algorithm is used to compute the power values on specific frequencies to compose a spectra. Using these two algorithms, the spatial MSE *L*_*S*−*MSE*_ and the frequency MSE *L*_*F*−*MSE*_ are defined for the generator:

(5) $$\begin{array}{r} L_{S-MSE}\left(G_{\theta_{G}}\right)=E_{\left(x,z\right)}\left[\frac{1}{C^{2}}||G\left(CSP\left(z\left(c\right)\right)\right)-CSP\left(x\left(c\right)\right)|{|}_{F}^{2}\right] \end{array}$$

(6) $$\begin{array}{r} L_{F-MSE}\left(G_{\theta_{G}}\right)=E_{\left(x,z\right)}\left[\frac{1}{N^{2}}||G\left(PSD\left(z\left(n\right)\right)\right)-PSD\left(x\left(n\right)\right)|{|}_{F}^{2}\right] \end{array}$$

where *CSP*(·) and *PSD*(·) are the CSP feature and PSD feature extractor, respectively. *c* is the channel of real EEG signals and the same of the generated EEG signals, *C* is the number of channels, *n* is the batch of real EEG signals and the same as that of the generated EEG signals, and *N* is the number of batches. For convenience, the TSF loss is computed by weighting three such MSE losses:

(7) $$\begin{array}{rc} L_{TSF-MSE}\left(G_{\theta_{G}}\right) & =\lambda_{T}\cdot L_{T-MSE}\left(G_{\theta_{G}}\right)+\lambda_{S}\cdot L_{S-MSE}\left(G_{\theta_{G}}\right)\\ +\lambda_{F}\cdot L_{F-MSE}\left(G_{\theta_{G}}\right), \end{array}$$

where λ_*T*_, λ_*S*_, λ_*F*_ are the weights of three such different MSE losses, respectively. Datasets with different sampling rates and sensitivities will obtain different weights, and, thus, the values of the weights will be determined by experiments.

In addition, to confirm that the EEG signals are temporally and spatially coherent, a regularization loss *L*_*TV*_(*G*_θ_*G*__) based on total variation is used in the generator:

(8) $$\begin{array}{r} L_{TV}\left(G_{\theta_{G}}\right)=\frac{1}{CT}\overset{C}{\underset{c=1}{\sum }}\overset{T}{\underset{t=1}{\sum }}||\nabla_{z}G_{\theta_{G}}{\left(z\right)}_{c,t}|| \end{array}$$

where ∇_*z*_(·) is the gradient calculator; the gradient regularization loss will encourage temporal and spatial coherence of the reconstruction. Combining Equations (3), (7), and (8), the overall joint reconstruction loss function is expressed as

(9) $$\begin{array}{r} \begin{array}{c} min\\ \theta_{G} \end{array}\begin{array}{c} max\\ \theta_{D} \end{array}L_{TSF-MSE}\left(G_{\theta_{G}}\right)+\lambda_{1}L_{WGAN}\left(D_{\theta_{D}},G_{\theta_{G}}\right)+\lambda_{2}L_{TV}\left(G_{\theta_{G}}\right) \end{array}$$

where λ_1_ and λ_2_ are the weights for controlling the trade-off among the WGAN adversarial loss, the TSF-MSE loss and the TV loss.

### 2.4. Network Structures

**Figure 1:**
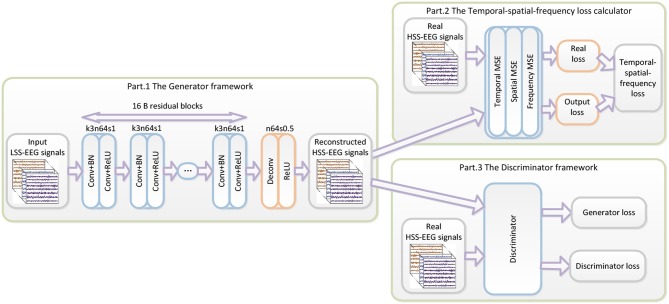
The architecture of the WGAN-EEG. The WGAN-EEG framework consists of three parts to reconstruct HSS-EEG signals from LSS-EEG signals. For the first part of the deep generator, “B residual blocks” with an identical layout are employed in the generator network. The second part of the WGAN-EEG framework is the TSF-MSE loss calculator. The third part of the WGAN-EEG is used to discriminate real HSS-EEG signals from generated HSS-EEG samples.

The proposed WGAN-EEG reconstruction framework is illustrated in Figure 1. The WGAN-EEG framework consists of three parts to reconstruct HSS-EEG signals from LSS-EEG signals. For the first part of the deep generator *G*_θ_*G*__, “B residual blocks” with an identical layout that was proposed by “Kaiming He” (He et al., 2016) are employed in the generator network. To facilitate the high sensitivity of EEG signals, 16 “B residual blocks” are applied to LSS-EEG signals to extract deep features for the generator. In each “B residual block,” following the common usage of the deep learning community, two convolutional layers with small 3*3 kernels, 1 stride, and 64 feature maps (k3n64s1) are followed by a batch-normalization layer (BN) and the ReLU activation function (Ioffe and Szegedy, 2015). To increase the sampling rate of the input EEG signals, the trained deconvolutional layer (stride = 0.5) is followed by “B residual blocks” to increase the sampling rate. In real-world application, the WGAN-EEG architecture is trained well to fit HSS-EEG signals before usage. In the usage scenario, the recorded LSS-EEG signals are incorporated into the well-trained architecture to reconstruct HSS-EEG signals to improve the sensitivity.

The second part of the WGAN-EEG framework is the TSF-MSE loss calculator, which is realized in Figure 1. The reconstructed output HSS-EEG signals *G*_θ_*G*__(*z*) from the generator *G*_θ_*G*__ and the ground truth HSS-EEG signals *x* are fed into the calculator to extract the CSP features and the PSD features. Then, using the extracted features, the TSF-MSE loss is computed by Equations (4), (5), (6). The reconstruction error computed by the loss function is then back-propagated to update the generator network's weights.

**Figure 2:**
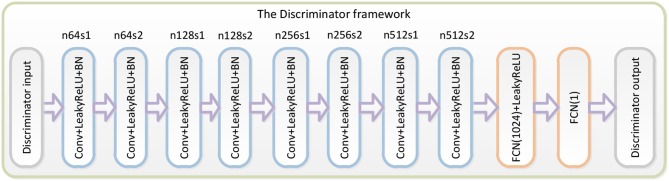
Details of the discriminator in the WGAN-EEG. We followed the architectural guidelines for the discriminator to use the LeakyReLU activation function and avoid max-pooling along the network. The discriminator network contains eight convolutional layers with an increasing number of filter kernels by a factor of 2. After eight convolutional layers, there are two FCN layers, of which the first layer has 1,024 outputs with the LeakyReLU activation function, and the second layer has a single output. Following the instructions of the WGAN, the discriminator of the WGAN-EEG has no sigmoid cross entropy layer.

The third part of the WGAN-EEG used to discriminate real HSS-EEG signals from generated HSS-EEG samples, the discriminator network *D*_θ_*D*__, is shown in Figure 2. Here, we followed the architectural guidelines for the discriminator to use the LeakyReLU activation function and avoid max-pooling along the network (Zhang et al., 2017). The discriminator network contains eight convolutional layers with an increasing number of filter kernels by a factor of 2. In fact, the convolutional kernels are increased from 64 to 512 kernels, and the stride is alternatively increased from 1 to 2 to reduce the EEG signal sampling rate when the number of features is doubled. In the discriminator, each convolutional layer is followed by a LeakyReLU activation function and a batch-normalization layer. After eight convolutional layers, there are two FCN layers, of which the first layer has 1,024 outputs with the LeakyReLU activation function, and the second layer has a single output. Following the instructions of the WGAN (Gulrajani et al., 2017), the discriminator of the WGAN-EEG has no sigmoid cross entropy layer.

The WGAN-EEG framework is trained by using EEG signal batches and applied on the entity of each signal trial. The details of training the WGAN have been described in the experiments.

## 3. Results

### 3.1. Experimental Datasets

To explore the feasibility and performance of the proposed algorithm, three EEG signal datasets with different sampling rates and sensitivities are applied to train and evaluate the proposed networks. Table 1 illustrates the details of these three different EEG datasets.

1. Action Observation (AO) dataset (Luo et al., 2018b): The AO dataset^1^ was collected from our previous research on different speed modes during AO. The EEG signals were acquired from the “NeuroScan SymAmp2” device with 64 channels, and the sampling rate and sensitivity were 250 Hz and 0.024 μV/bit, respectively. In this dataset, six subjects were invited to observe a robot's actions at four different speeds. Thus, the dataset had 24 subsets for each subject in each AO speed mode. Each subset contained 384 trials with 192 trials of left leg movements and 192 trials of right leg movements for a binary classification, and each trial lasted 5 s. To train the GAN/WGAN, a “leave-one-rest” strategy is used for training. In our pre-training experiments, more signals caused a problem of over-fitting and a large time complexity for GAN/WGAN training. Since 13 subsets containing 4,992 trials were enough to obtain the best performance, we left one subset and randomly selected 13 subsets from the remaining 23 subsets for training; the left subset was reconstructed after obtaining the well-trained GAN/WGAN. Therefore, all 24 subsets were reconstructed through 24 rounds of the above procedure. Because the AO dataset was acquired at a sampling rate of 250 Hz, we down-sampled all trials of EEG samples to the sampling rate of 125 Hz for the sake of sampling rate reconstruction.
2. Grasp and Lift (GAL) dataset (Luciw et al., 2014): The GAL dataset^2^ recorded EEG signals while the subjects grasped and lifted an object. The EEG signals were acquired using the “BrainAmp” device with 32 channels, and the sampling rate and sensitivity were 500 Hz and 0.1 μV/bit, respectively. In this dataset, 12 subjects executed six movements for 1,560 trials, and each trial lasted 0.5 s; thus, the classification of EEG signals contained six categories. To train the GAN/WGAN, a “leave-one-rest” strategy is used for training. The 9,360 trials carried out by six subjects were enough to train the GAN/WGAN, and we thus left one subject's signals and randomly selected six subjects' signals from the remaining 11 subjects' signals for training; the left subjects' signals were reconstructed after obtaining the well-trained GAN/WGAN. Therefore, all 12 subjects' signals were reconstructed through 12 rounds of the above procedure. In the experiment, to validate the reconstruction of the sampling rate, all signals were down-sampled to a sampling rate of 250 Hz.
3. Motor Imagery (MI) dataset (Tangermann et al., 2012): The MI dataset^3^ was from the “BCI competition IV dataset 2a.” Nine subjects participated in the MI experiment during which EEG signals were recorded while the subject imagined his/her own leg, foot, and tongue movements, and each trial lasted for 4 s. There were 22 channels, and the sampling rate and sensitivity were 250 Hz and 100 μV/bit, respectively. In this dataset, nine subjects executed four motor imagery tasks, and each subject had 576 trials of EEG signals for a four categories for classification.To train the GAN/WGAN, a “leave-one-rest” strategy is used for training. The 4,032 trials carried out by seven subjects were enough to train the GAN/WGAN, and we thus left one subject's signals and randomly selected seven subjects' signals from the remaining eight subjects' signals for training; the left subjects' signals were reconstructed after obtaining the well-trained GAN/WGAN. Therefore, all nine subsets were reconstructed through nine rounds of the above procedure. For the same purpose, all trials of EEG signals were down-sampled at a sampling rate of 125 Hz.

**Table 1:** Details of the three different EEG datasets.

**Datasets**	**Action observation**	**Grasp and lift**	**Motor imagery**
Sampling rate/s	250	500	250
Sensitivity/bit	0.024 μV/bit	0.1 μV/bit	100 μV/bit
Channels	64	32	22
Classification categories	2	6	4
Subject number	6	12	9
Trials/Subject	384	576	1,560
Trial duration/s	5 s	0.5 s	4 s
Device	NeuroScan SymAmp2	BrainAmp	Unknown

### 3.2. Training Details

In the training procedure, we trained six models using the GAN/WGAN framework within three different datasets. All down-sampled training EEG samples were fed into the generator, and the real training EEG samples were fed into the discriminator. The generated EEG samples and the real EEG samples were discriminated by the TSF-MSE loss function to update the generator and the discriminator for solving the min-max problem. Because the AO dataset and the GAL dataset have high sampling rates and a high number of channels, models for these two datasets were trained over 30 epochs. However, the MI dataset has a lower sampling rate and fewer channels, and, therefore, this dataset was trained over 20 epochs. Each epoch traverses all the data in the corresponding dataset. According to the different devices used to record EEG signals, the generators of the GAN/WGAN frameworks were specified by different scopes of generation for different datasets. We specified the generation scopes of [−40, 40 μV], [−50, 50 μV], and [−100, 100 μV] for the AO dataset, the GAL dataset, and the MI dataset, respectively.

**Figure 3:**
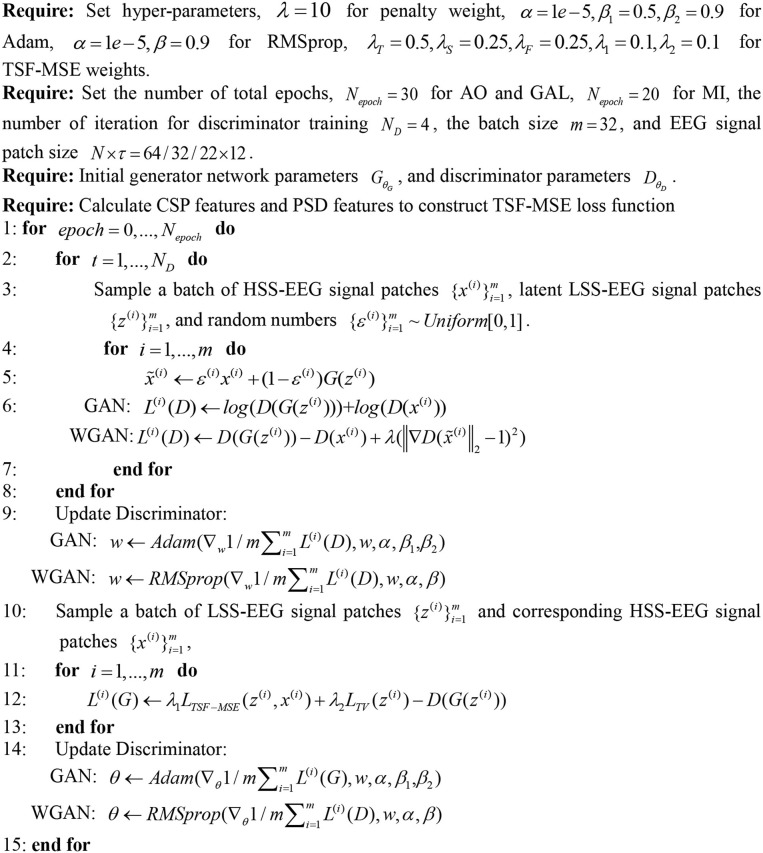
The optimization procedure for the GAN/WGAN. Following the instructions of the GAN/WGAN frameworks, the Adam optimizer's hyperparameters are set as α = 1*e*−5, β_1_ = 0.5, β_2_ = 0.9, and the RMSprop optimizer's hyperparameters are set as α = 1*e*−5, β = 0.9. The hyperparameter for the gradient penalty is set as λ = 10 according to the suggestion in the reference. The hyperparameters in the TSF-MSE loss function and the joint reconstruction are set as λ_*T*_ = 0.5, λ_*S*_ = 0.25, λ_*F*_ = 0.25, λ_1_ = 0.1, λ_2_ = 0.1 according to our experimental experience. The optimization processes for the GAN and the WGAN are similar, except some places are changed to the corresponding optimizer and the loss functions.

In our experiments, we randomly extracted pairs of signal patches from down-sampled EEG signals and real EEG signals as our training inputs and labels. The patch size is N*τ, where *N* is the channel number for different datasets, and τ is the EEG samples from the temporal domain. Since the limited trials of EEG signals (<500 trials for one subject) and smaller values of τ will construct more accurate sequential relationships for the EEG signals, following our previous research (Luo et al., 2018a), we cropped a minimal length for the training of the deep neural network. According to the pre-experiment, we set τ = 12 to satisfy the minimal length for the convolution in the GAN/WGAN architecture. In the optimization of the generator and the discriminator, according to current research (Basu et al., 2018), the GAN models were optimized by the Adam algorithm (Basu et al., 2018), and the WGAN models were optimized by the RMSprop algorithm (Mukkamala and Hein, 2017). The optimization procedure for the GAN/WGAN architectures is shown in Figure 3. The mini-batch size was set to 32. Following the instructions of the GAN/WGAN frameworks (Goodfellow et al., 2014; Gulrajani et al., 2017), the Adam optimizer's hyperparameters were set as $\alpha =10^{-5},\beta_{1}=0.5,\beta_{2}=0.9$, and the RMSprop optimizer's hyperparameters were set as α = 10^−5^, β = 0.9. The hyperparameter for the gradient penalty of WGAN framework was set as λ = 10 according to the suggestion in the reference (Gulrajani et al., 2017). The hyperparameters for the SRGAN/SRGAN frameworks in Equation (9) were set as $\lambda_{1}=10^{-3}$ and $\lambda_{2}=2*10^{-8}$ by the suggestions of reference (Ledig et al., 2017). The hyperparameters in the TSF-MSE loss function of Equation (7) and the joint reconstruction were set of different values according to the experimental experience of each reconstruction round, and the average values with standard deviations of all parameters in three datasets are given in Table 2. The optimization processes for the GAN framework and the WGAN framework are similar; however, some places are changed to the corresponding optimizer and the loss functions (see Figure 3).

**Table 2:** The hyperparamter λ_*T*_, λ_*S*_, λ_*F*_ tuning of the novel TSF-MSE loss function for all experiments.

**Reconstruction**	**λ_*T*_**	**λ_*S*_**	**λ_*F*_**
*AO*−>*AO*	0.46 ± 0.12	0.23 ± 0.06	0.30 ± 0.09
*GAL*−>*GAL*	0.44 ± 0.13	0.21 ± 0.08	0.35 ± 0.06
*MI*−>*MI*	0.53 ± 0.16	0.20 ± 0.11	0.27 ± 0.03
*GAL*−>*AO*	0.47 ± 0.12	0.18 ± 0.09	0.31 ± 0.07
*MI*−>*AO*	0.41 ± 0.13	0.31 ± 0.08	0.27 ± 0.09
*AO*−>*GAL*	0.50 ± 0.14	0.22 ± 0.09	0.33 ± 0.06
*MI*−>*GAL*	0.52 ± 0.12	0.22 ± 0.08	0.25 ± 0.08
*AO*−>*MI*	0.60 ± 0.18	0.31 ± 0.09	0.07 ± 0.01
*GAL*−>*MI*	0.58 ± 0.17	0.33 ± 0.08	0.06 ± 0.01

The GAN/WGAN frameworks were implemented in Python 2.7 with the Tensorflow 1.8 library. Two NVIDIA 1080Ti GPUs were used in this study.

### 3.3. Network Convergence

**Figure 4:**
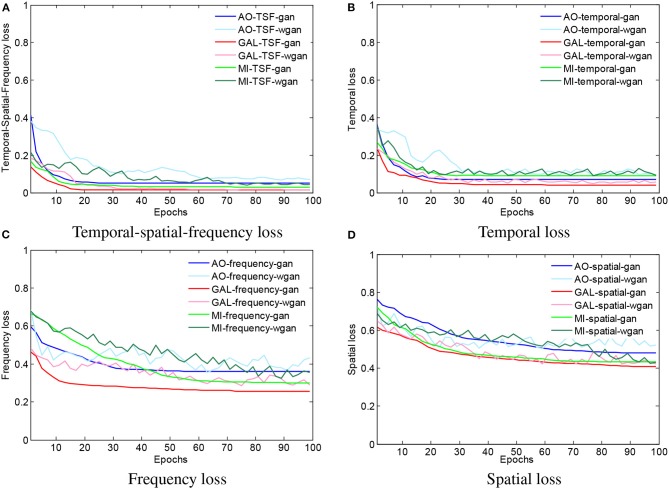
The averaged MSE and Wasserstein distance estimations for training the GAN/WGAN. In the four figures, all of the iterative curves decreased rapidly within the first 10 epochs (each epoch contains 10 errors recording), and the initial decreases indicated that these two metrics are positively correlated for the EEG signal reconstruction. However, for each dataset or using GAN/WGAN frameworks, the loss results of TSF-MSE are lower than the loss results of conventional temporal, frequency, and spatial MSE. In addition, of these four losses, the WGAN frameworks oscillate in the convergence process, while the GAN frameworks are smoothed in the convergence process. **(A)** Temporal-spatial-frequency loss, **(B)** Temporal loss, **(C)** Frequency loss, **(D)** Spatial loss.

To visualize the convergence of the GAN/WGAN frameworks, the conventional temporal MSE, frequency MSE, spatial MSE, the proposed TSF-MSE losses, and the Wasserstein distance for validation of three different datasets were computed according to Equations (2), (3), (4), and (5). Figure 4 shows the averaged temporal MSE, frequency MSE, spatial MSE, and TSF-MSE losses vs. the number of epochs for different datasets within the GAN/WGAN frameworks.

From Figures 4A–D, for a given framework and dataset, we have compared the variations and differences between the conventional temporal MSE, frequency MSE, spatial MSE, and our proposed TSF-MSE. In the four figures, all of the iterative curves are shown to have decreased rapidly within the first 10 epochs (each epoch contains 10 error recordings), and the initial decreases indicated that these two metrics are positively correlated for the EEG signal reconstruction. However, for each dataset or when using GAN/WGAN frameworks, the loss results of TSF-MSE were lower than the loss results of conventional temporal MSE, frequency MSE, and spatial MSE. In addition, of these four losses, the WGAN frameworks oscillated in the convergence process, while the GAN frameworks smoothed in the convergence process. Comparing the oscillation of losses, the TSF loss exhibited varied smoothing for the WGAN framework compared to the GAN framework for each dataset. These observations of network convergence suggested that the conventional MSE losses and our proposed TSF-MSE loss have different focuses within the GAN/WGAN frameworks. By applying the generators, the difference between conventional MSE losses and our proposed TSF-MSE loss will be further revealed in the reconstructed EEG signals.

**Figure 5:**
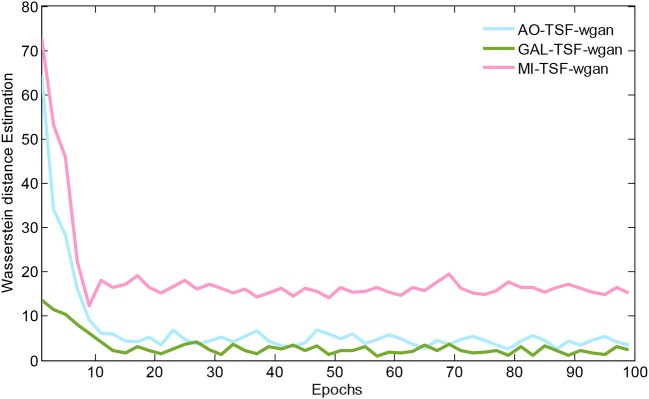
The Wasserstein distance estimation vs. the number of epochs for three different datasets. The plotted Wasserstein values are estimated by the definition of −*E*_*x*~_*P*__*H*__[*D*_θ_*D*__(*x*)]+*E*_*z*~_*P*__*L*__[*D*_θ_*D*__(*G*_θ_*G*__(*z*))] in Equation (3). For the curves of these three datasets, we note that the Wasserstein distance we computed is a surrogate that has not been normalized by the total number of EEG signal samplings, and, therefore, the curves would have decreased to close to zero after 100 epochs by using the normalization for the EEG signals.

Figure 5 illustrates the Wasserstein distance estimation vs. the number of epochs for three different datasets. The plotted Wasserstein values were estimated by the definition of −*E*_*x*~_*P*__*H*__[*D*_θ_*D*__(*x*)]+*E*_*z*~_*P*__*L*__[*D*_θ_*D*__(*G*_θ_*G*__(*z*))] in Equation (3). From the figure, we have found a reduction in the Wasserstein distances as the number of epochs increased, but different datasets have different decay rates of the reducing Wasserstein distance. For the curves of the three datasets, we noted that the Wasserstein distance we computed is a surrogate that has not been normalized by the total number of EEG signal samplings, and, therefore, the curves would have decreased to close to zero after 100 epochs by using the normalization for the EEG signals.

### 3.4. Reconstruction Results

**Figure 6:**
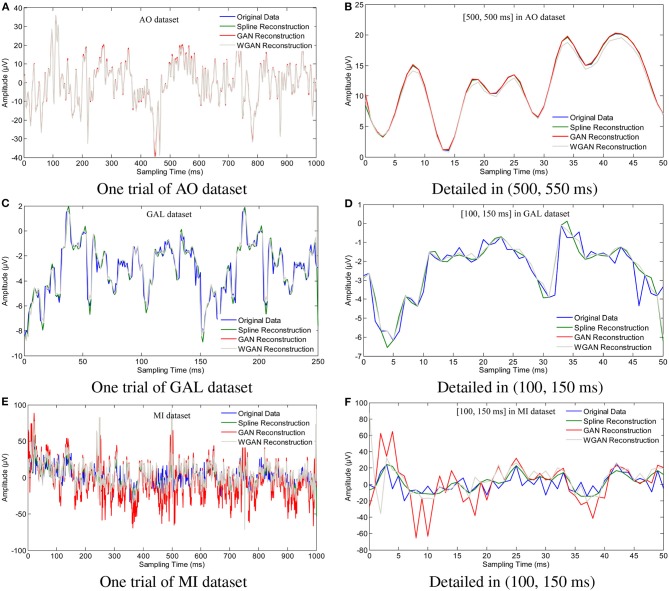
Sampling rate reconstruction by the same sensitivity GAN/WGAN frameworks. Sampling rate and sensitivity reconstruction by the same sensitivity GAN/WGAN frameworks. The reconstruction results of one trial for AO dataset, GAL dataset, and MI dataset. Meanwhile, the detailed reconstruction results in (500, 550 ms) of AO datasets and (100, 150 ms) of GAL and MI datasets are also given. **(A)** One trial of AO dataset, **(B)** Detailed in (500, 550 ms), **(C)** One trial of GAL dataset, **(D)** Detailed in (100, 150 ms), **(E)** One trial of MI dataset, **(F)** Detailed in (100, 150 ms).

**Figure 7:**
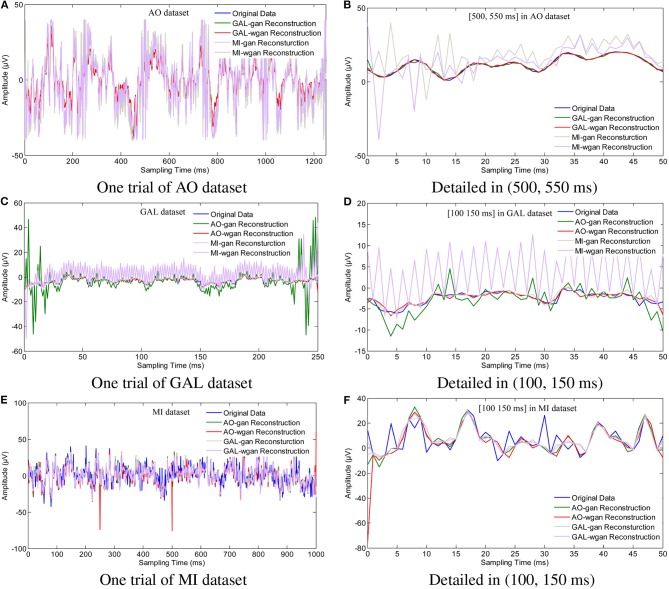
Sampling rate reconstruction by different sensitivity GAN/WGAN frameworks. Sampling rate and sensitivity reconstruction by different sensitivity GAN/WGAN frameworks. The reconstruction results of one trial for AO dataset, GAL dataset, and MI dataset. Meanwhile, the detailed reconstruction results in (500, 550 ms) of AO datasets and (100, 150 ms) of GAL and MI datasets are also given. **(A)** One trial of AO dataset, **(B)** Detailed in (500, 550 ms), **(C)** One trial of GAL dataset, **(D)** Detailed in (100, 150 ms), **(E)** One trial of MI dataset, **(F)** Detailed in (100, 150 ms).

**Figure 8:**
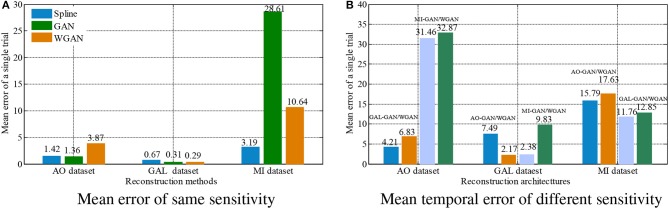
Statistical mean temporal error comparison between the same and different sensitivity GAN/WGAN frameworks.The high-sensitivity EEG signals' GAN/WGAN frameworks reconstruct the low sensitivity EEG signals well, such as the AO and GAL data reconstructed by the MI GAN/WGAN frameworks. However, the low sensitivity EEG signals' GAN/WGAN models cannot reconstruct accurate high-sensitivity EEG signals, such as MI data reconstructed by the AO and GAL GAN/WGAN frameworks. **(A)** Mean error of same sensitivity, **(B)** Mean temporal error of different sensitivity.

**Figure 9:**
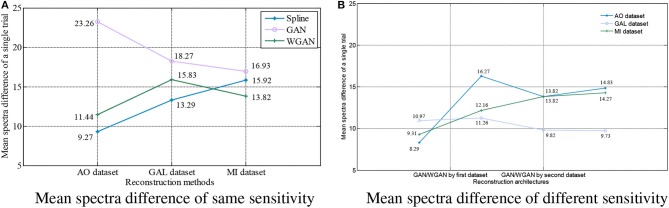
Statistical mean spectra difference comparison between the same and different sensitivity GAN/WGAN frameworks. For the reconstructions of the same sensitivity, the mean spectra results have shown that WGAN architectures outperform than GAN architectures. As for the reconstructions of different sensitivity, we found that higher sensitivity models brought lower spectra difference, while lower sensitivity models brought higher spectra difference. **(A)** Mean spectra difference of same sensitivity, **(B)** Mean spectra difference of different sensitivity.

**Figure 10:**
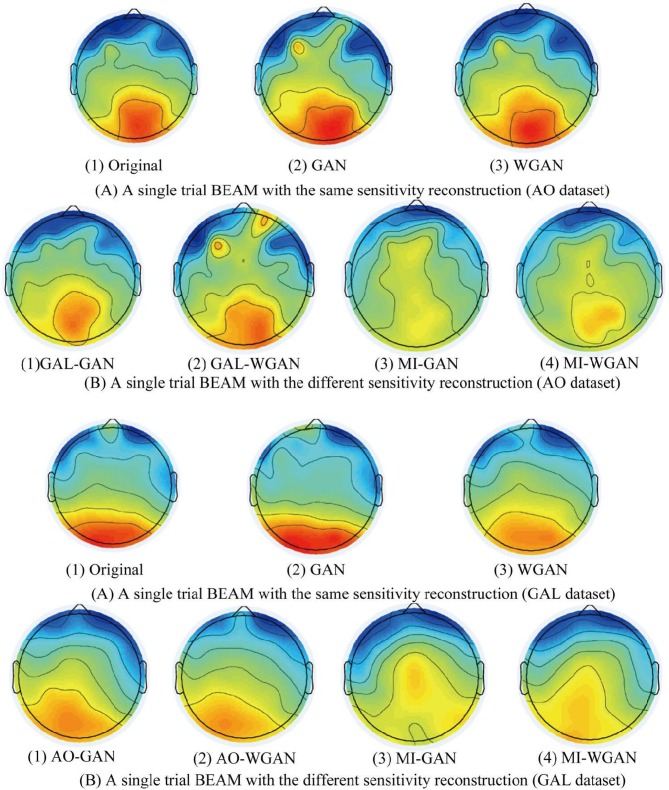
A single reconstruction trial BEAM on 12 Hz comparison between the same and different sensitivity GAN/WGAN frameworks. For the reconstructions of the same sensitivity, the BEAM results have shown that WGAN architectures outperform GAN architectures. As for the reconstructions of different sensitivity, we have found that high-sensitivity models bring more distinct ERS/ERD phenomenon on brain electrical activity mappings (BEAMs), while low-sensitivity models bring less distinct ERS/ERD phenomenon on BEAMs. **(A)** BEAMs of AO datasets with same and different sensitivity reconstruction. **(B)** BEAMs of GAL datasets with same and different sensitivity reconstruction.

To show the reconstruction effects of the GAN/WGAN frameworks with our proposed TSF-MSE loss function, we considered two different aspects of the reconstruction results. The first one was the sampling rate reconstruction by the same sensitivity signals' GAN/WGAN frameworks, which is shown in Figure 6. The second one was the sensitivity rate reconstruction by the different sensitivity signals' GAN/WGAN frameworks, which is shown in Figure 7. Since the proposed reconstruction method used a novel TSF-MSE loss function for the training of GAN/WGAN architectures, the statistical temporal, frequency, and spatial results were also compared between the original signals and the reconstructed signals. Figures 8–10 illustrated the mean temporal error, mean spectra difference, and brain electrical activity mapping on 12 Hz of a single trial compared with the original EEG signals and all reconstructed EEG signals.

To plot the reconstruction results of different models and situations, we chose the same trial from each dataset for the comparison experiments. Because the number of channels differs for each dataset, we choose the “*FPz*” channel for the experiments to plot the figures. In addition, as one trial over a long period of time will hide some details of the reconstruction signals, we chose the 50 ms range of (500 and 550 ms) for the AO and MI datasets and the 50 ms range of (100 and 150 ms) for the GAL dataset to plot the details of the reconstruction results. From the reconstruction results by the same details shown in Figure 6, we have found that the signals' proximity between the reconstructed data and the original data decreased in the following order for the three datasets: AO > GAL > MI. The difference between the GAN framework and the WGAN framework cannot be realized at the signal level. In the figures shown in Figure 7, the high sensitivity EEG signals' GAN/WGAN frameworks reconstructed the low sensitivity EEG signals well, such as the AO and GAL data reconstructed by the MI GAN/WGAN frameworks. However, the low sensitivity EEG signals' GAN/WGAN models cannot reconstruct accurate high sensitivity EEG signals, such as MI data reconstructed by the AO and GAL GAN/WGAN frameworks.

For the statistical results in Figures 8–10, we have found that excepting for the temporal errors, reconstructed EEG signals show the same regulations on frequency and spatial features. For the reconstructions of the same sensitivity, the mean spectra results have shown that WGAN architectures outperform than GAN architectures, so do the brain electrical activity mapping (BEAM) results for reconstructions of the same sensitivity. As for the reconstructions of different sensitivity, we have found that higher sensitivity models bring lower spectra difference and more distinct ERS/ERD phenomenon on BEAMs, while lower sensitivity models bring higher spectra difference and less distinct ERS/ERD phenomenon on BEAMs.

### 3.5. Classification Results

In fact, the qualitative analysis could not yield promising insight regarding HSS-EEG signals reconstructed by LSS-EEG signals. Hence, a quantitative analysis was applied to explore the performance of reconstructed EEG signals. In this paper, because the AO dataset corresponded to action observation, the GAL dataset corresponded to action execution, and the MI dataset corresponded to motor imagery, these three datasets caused the same event-related desynchronization/event-related synchronization (ERD/ERS) phenomenon, which can be classified by filter bank common spatial patterns (FBCSP) and a support vector machine (SVM) (Luo et al., 2018a,b). The ERS/ERD phenomenon from EEG signals is common on three motor-related datasets, and such phenomena are usually used for the motor-based BCI. Therefore, the ERS/ERD phenomenon will be the key index with which to measure the performance of BCI system by EEG signals. This study thus selected the ERS/ERD phenomenon from EEG signals as a quantitative measure, and FBCSP features with an SVM classifier were applied to explore the performances of the original signals and the reconstructed signals. For comparison with different models and different sensitivities, there were several hyperparameters for the FBCSP features, SVM classifier, and deep learning classifier:

1. Because all three datasets contain the ERD/ERS phenomenon, which is detected on the band of [8, 30 Hz], the filter bank strategy is used to divide the whole band to obtain universality for different subjects. In this study, the width and overlapping ratio were set to 4 and 2 Hz for the filter bank dividing, as shown in Table 3. After the EEG signals are filtered by the optimal filter bank, the CSP algorithm was included to extract FBCSP features (Ang et al., 2012).
2. The CSP algorithm (Ang et al., 2012) is presented to every filter result to extract the optimal spatial features by computing a problem of maximizing the power ratio for different AO/AE/MI tasks. Then, the maximizing power ratio is computed by the singular value decomposition (SVD) algorithm to obtain eigenvalues and eigenvectors. Because different datasets have EEG signals from different channels, the number of eigenvalues used for constructing the CSP spatial vector were set to *m* = 8, *m* = 4, and *m* = 4 for the AO dataset, the GAL dataset, and the MI dataset, respectively.
3. In the classification, the SVM classifier was issued to classify the extracted FBCSP features from three different datasets. To overcome the non-stationary and nonlinear characteristics of EEG signals, the linear kernel with hyperparameters was set to *c* = 0.01 and *g* = 2 for the classifiers for all datasets. To compare the classification performance for both the original data and the reconstructed data, an 8*8 cross-validation strategy was applied to each dataset, and the average classification results were recorded.
4. In order to validate the performance improvement of reconstructed signals, a convolutional neural networks based deep learning model “FBCSPNet” from reference (Schirrmeister et al., 2017) was introduced to compare the classification performance between original signals and reconstructed signals. Experimental parameters were set as the same from the reference for AO/GAL/MI datasets.

**Table 3:** The optimal division of bandpass filters.

Sub-bands	*fb*_1_	*fb*_2_	*fb*_3_	*fb*_4_	*fb*_5_
Frequency (Hz)	[8, 12]	[10, 14]	[12, 16]	[14, 18]	[16, 20]
Sub-bands	*fb*_6_	*fb*_7_	*fb*_8_	*fb*_9_	*fb*_10_
Frequency (Hz)	[18, 22]	[20, 24]	[22, 26]	[24, 28]	[26, 30]

Classification results for the sampling rate reconstruction by the same sensitivity signals' GAN/WGAN frameworks are shown in Tables 4–6 for AO dataset, GAL dataset, and MI dataset, respectively. In addition, classification results for the sensitivity rate reconstruction by the different sensitivity signals' GAN/WGAN frameworks are shown in Tables 7–9 for AO datset, GAL dataset, and MI dataset, respectively. In all tables, the results are presented by classification accuracy forms, and a paired *t*-test statistical technique was used to detect whether the reconstructed EEG signals significantly outperform than the original EEG signals. P-value of the t-test statistics are provided in the tables, and ^*^p <0.05 and ^*^^*^p <0.01 represent the results compared among two columns are significantly different and extremely significantly different.

**Table 4:** Classification results of GAN/WGAN frameworks for the sampling rate reconstruction of the same sensitivity signals in AO dataset.

**Subjects**	**Original data (a)**	**Spline data (b)**	**GAN up-sampling (c)**	**WGAN up-sampling (d)**
AO-1	62.83	58.72	64.51	**69.52**
AO-2	53.12	51.67	52.83	**55.61**
AO-3	70.31	68.92	**73.96**	72.66
AO-4	75.26	71.78	73.96	**80.99**
AO-5	59.12	58.27	61.28	**64.22**
AO-6	74.22	70.30	73.96	**76.82**
AO-7	55.43	54.28	**60.72**	59.83
AO-8	58.27	55.42	**59.82**	59.73
AO-9	77.08	76.07	83.85	**84.37**
AO-10	68.49	68.78	**75.26**	70.57
AO-11	79.17	74.64	87.76	**89.85**
AO-12	73.44	66.15	66.67	**75.53**
AO-13	55.83	59.42	61.42	**63.49**
AO-14	61.82	55.23	63.83	**65.72**
AO-15	55.49	53.82	56.29	**58.73**
AO-16	62.42	57.59	63.86	**65.82**
AO-17	64.58	57.81	58.59	**67.44**
AO-18	53.28	53.28	55.87	**57.89**
AO-19	52.82	51.87	55.89	**57.63**
AO-20	63.83	62.57	**68.59**	67.83
AO-21	70.31	61.98	61.98	**72.91**
AO-22	57.55	55.73	57.29	**59.37**
AO-23	61.83	58.89	**63.82**	63.58
AO-24	59.27	58.73	62.82	**63.93**
AVG	63.57	60.91	65.41	67.67
*T*-test	–	a vs. b	c vs. a	d vs. a
*p*-value	–	**p <0.01	*p <0.05	**p <0.01

*The bold text is the best performance*.

**Table 5:** Classification results of GAN/WGAN frameworks for the sampling rate reconstruction of the same sensitivity signals in GAL dataset.

**Subjects**	**Original data (a)**	**Spline data (b)**	**GAN up-sampling (c)**	**WGAN up-sampling (d)**
GAL-1	69.23	68.71	72.81	**75.63**
GAL-2	65.06	65.42	**68.93**	68.72
GAL-3	74.04	71.69	79.83	**80.54**
GAL-4	59.17	58.66	**62.82**	61.93
GAL-5	79.94	74.48	**83.61**	82.83
GAL-6	69.04	69.76	74.63	**75.59**
GAL-7	74.33	68.79	**68.67**	68.27
GAL-8	79.81	77.62	79.82	**80.42**
GAL-9	64.04	63.48	72.24	**72.68**
GAL-10	65.38	65.48	74.81	**75.61**
GAL-11	63.21	62.52	**72.60**	72.07
GAL-12	74.10	72.42	**72.85**	72.36
AVG	69.78	68.25	73.63	73.89
*T*-test	–	a vs. b	c vs. a	d vs. a
*p*-value	–	*p <0.05	*p <0.05	*p <0.05

*The bold text is the best performance*.

**Table 6:** Classification results of GAN/WGAN frameworks for the sampling rate reconstruction of the same sensitivity signals in the MI dataset.

**Subjects**	**Original data (a)**	**Spline data (b)**	**GAN up-sampling (c)**	**WGAN up-sampling (d)**
MI-1	60.59	57.82	61.62	**62.81**
MI-2	70.31	68.58	71.18	**73.35**
MI-3	53.47	53.82	54.93	**56.81**
MI-4	59.38	56.71	**61.83**	60.62
MI-5	72.22	70.49	73.88	**76.57**
MI-6	67.36	65.73	68.72	**68.81**
MI-7	56.25	55.41	**58.83**	57.61
MI-8	57.81	56.43	**58.81**	57.69
MI-9	60.42	58.67	**62.73**	61.85
AVG	61.98	60.41	63.61	64.01
*T*-test	–	a vs. b	c vs. a	d vs. a
*p*-value	–	**p <0.01	**p <0.01	**p <0.01

*The bold text is the best performance*.

**Table 7:** Classification results for the sensitivity rate reconstruction of AO dataset by the different sensitivity signals' GAN/WGAN frameworks.

**Subjects**	**Original data (a)**	**GAL-WGAN (b)**	**GAL-GAN (c)**	**MI-WGAN (d)**	**MI-GAN (e)**
**AO dataset reconstructed by the gal and mi models**					
AO-1	62.83	**64.81**	64.57	60.73	61.82
AO-2	53.12	55.82	**56.18**	53.27	52.63
AO-3	70.31	72.13	**73.18**	69.53	70.83
AO-4	75.26	73.44	**75.78**	67.71	69.53
AO-5	59.12	61.73	**62.16**	58.83	56.76
AO-6	74.22	76.56	**77.87**	76.82	75.26
AO-7	55.43	**57.61**	56.89	55.43	54.68
AO-8	58.27	**59.46**	58.83	56.27	56.29
AO-9	77.08	76.56	**77.34**	72.14	73.44
AO-10	68.49	72.66	70.57	**75.26**	71.09
AO-11	79.17	83.07	**83.85**	78.91	81.51
AO-12	**73.44**	71.62	72.57	70.57	66.93
AO-13	55.83	56.73	**56.94**	54.87	55.16
AO-14	61.82	62.73	**63.81**	60.57	60.81
AO-15	55.49	**56.81**	56.43	55.36	55.61
AO-16	62.42	63.55	**64.31**	61.28	61.37
AO-17	**64.58**	61.72	60.03	55.47	56.25
AO-18	53.28	53.89	**53.61**	52.13	52.28
AO-19	52.82	53.61	**54.18**	52.36	53.17
AO-20	63.83	**64.81**	63.76	62.67	62.89
AO-21	**68.72**	63.59	62.18	57.62	58.73
AO-22	57.55	59.11	59.55	60.42	**61.46**
AO-23	61.83	62.81	**63.75**	62.19	61.68
AO-24	59.27	60.73	**60.81**	59.36	59.81
AVG	63.51	64.40	64.55	62.07	62.08
*T*-test	–	b vs. a	c vs. a	d vs. a	e vs. a
*p*-value	–	*p <0.05	*p <0.05	*p* = 0.0738	*p <0.05

*The bold text is the best performance*.

**Table 8:** Classification results for the sensitivity rate reconstruction of GAL dataset by the different sensitivity signals' GAN/WGAN frameworks.

**Subjects**	**Original data (a)**	**AO-WGAN (b)**	**AO-GAN (c)**	**MI-WGAN (d)**	**MI-GAN (e)**
**GAL dataset reconstructed by the ao and mi models**					
GAL-1	69.23	**73.53**	72.76	72.56	70.19
GAL-2	**65.06**	63.21	62.88	61.86	61.03
GAL-3	**74.04**	59.74	66.03	61.99	58.59
GAL-4	59.17	59.74	**66.03**	61.99	58.59
GAL-5	79.94	76.28	**80.96**	76.15	77.18
GAL-6	69.04	**73.33**	72.56	73.21	76.35
GAL-7	**74.33**	69.77	66.25	64.82	66.91
GAL-8	79.81	78.53	74.17	**81.92**	79.49
GAL-9	64.04	65.58	65.58	64.30	**67.63**
GAL-10	65.38	62.76	62.24	60.64	**67.50**
GAL-11	63.21	85.13	85.00	**85.51**	85.38
GAL-12	74.10	**76.47**	72.76	69.94	73.72
AVG	69.78	70.34	**70.60**	69.57	70.21
*T*-test	–	b vs. a	c vs. a	d vs. a	e vs. a
*p*-value	–	*p* = 0.821	*p* = 0.731	*p* = 0.937	*p* = 0.870

*The bold text is the best performance*.

**Table 9:** Classification results for the sensitivity rate reconstruction of MI dataset by the different sensitivity signals' GAN/WGAN frameworks.

**Subjects**	**Original data (a)**	**AO-WGAN (b)**	**AO-GAN (c)**	**GAL-WGAN (d)**	**GAL-GAN (e)**
**MI dataset reconstructed by the gal and ao**					
**GAN/wgan models**					
MI-1	60.59	61.63	59.90	58.33	**67.53**
MI-2	70.31	71.70	69.10	**75.00**	73.26
MI-3	53.47	**58.16**	54.17	53.99	55.03
MI-4	59.38	**68.58**	68.58	64.41	66.67
MI-5	72.22	68.92	**76.22**	73.09	66.67
MI-6	67.36	**70.49**	70.49	69.62	68.75
MI-7	56.25	58.68	**73.44**	58.85	60.07
MI-8	57.81	54.34	54.51	**63.54**	56.60
MI-9	**60.42**	57.12	57.99	56.08	55.90
AVG	61.98	63.29	**64.93**	63.66	63.39
*T*-test	–	b vs. a	c vs. a	d vs. a	e vs. a
*p*-value	–	*p* = 0.380	*p* = 0.215	*p* = 0.175	*p* = 0.215

*The bold text is the best performance*.

Tables 4–6 illustrate the up-sampling classification results compared with the original data, the spline reconstructed data, the GAN reconstructed data, and the WGAN reconstructed data. Among the three datasets, we have found that the WGAN reconstructed data achieved the best classification performance. In the AO dataset, the WGAN reconstructed signals achieved the best classification accuracy (67.67%), which was higher than those of the original data (63.57%), the spline reconstructed data (60.91%), and the GAN reconstructed data (65.41%). In the GAL dataset, the WGAN reconstructed signals achieved the best classification accuracy (73.89%), which was higher than those of the original data (69.78%), the spline reconstructed data (68.25%), and the GAN reconstructed data (73.63%). In the MI dataset, the WGAN reconstructed signals achieved the best classification accuracy (64.01%), which was higher than those of the original data (61.98%), the spline reconstructed data (60.41%), and the GAN reconstructed data (63.61%).

From the *t*-test statistical results that computed compared signals, the reconstructed GAN/WGAN model signals exhibited significant improvement of classification, producing a better performance than the original signals, while spline reconstructed signals exhibited significant reduction of classification performance, lower that of the original signals. The significant improvement and reduction are presented for all AO/GAL/MI datasets (^*^p <0.05). Specifically for the WGAN model in AO dataset and GAN/WGAN model in MI dataset, the classification performances presented were extremely significant (^*^^*^p <0.01). Therefore, we have concluded that the GAN/WGAN models with proposed TSF-MSE loss function showed a significant improvement for reconstructing EEG signals with the same sensitivity.

Tables 7–9 give the classification results compared with the GAN/WGAN models trained with different sensitivities. Table 7 gives the classification results of the AO data reconstructed by the GAL/MI trained GAN/WGAN models. Table 8 gives the classification results of the GAL data reconstructed by the AO/MI trained GAN/WGAN models. Table 9 gives the classification results of the MI data reconstructed by the AO/GAL trained GAN/WGAN models. For the AO dataset, signals reconstructed by the GAL-GAN model achieve the best average classification accuracy (64.55%), which was higher than those of the original data (63.51%) and the data reconstructed by the GAL-WGAN (64.40%), the MI-WGAN (62.07%), and the MI-GAN (62/08%). For the GAL dataset, signals reconstructed by the AO-GAN model achieve the best average classification accuracy (70.60%), which is higher than those of the original data (69.78%) and the data reconstructed by the AO-WGAN (70.34%), the MI-WGAN (69.57%), and the MI-GAN (70.21%). For the MI dataset, signals reconstructed by the AO-GAN model achieved the best average classification accuracy (64.93%), which was higher than those of the original data (61.98%) and the data reconstructed by the AO-WGAN (63.29%), the MI-WGAN (63.66%), and the MI-GAN (63.39%). The GAN model performed better than the WGAN model for reconstructing EEG signals by different sensitivities, and LSS-EEG signals reconstructed by HSS-EEG models will increase the sampling rate and sensitivity of signals, which will increase the classification performance.

From the *t*-test statistical results that computed between compared signals, the AO dataset reconstructed signals by GAL-WGAN and GAL-GAN, showing significant improvement of classification performance than the original signals (*p <0.05), while other datasets reconstructed signals showed no significant performance compared to the original signals(*p > 0.05). In addition, AO dataset reconstructed signals by MI-GAN a classification performance that was significantly worse than the original signals (*p <0.05). Therefore, we have concluded that the GAN/WGAN models with proposed TSF-MSE loss function showed significant performance improvement with enough data and no significant performance improvement without enough data for reconstructing EEG signals with the same sensitivity. Besides, if there is a large gap of sensitivity between two EEG signals datasets, the lower sensitivity based GAN model will cause significant worse performance of reconstructing high sensitivity signals to low sensitivity signals (such as MI-GAN applied to AO dataset).

Since this study has proposed a novel loss function to build the GAN/WGAN architectures for reconstructions, we have also compared the mean classification accuracy between temporal-MSE based GAN/WGAN architectures and TSF-MSE based GAN/WGAN architectures. Due to the single spatial-MSE and frequency-MSE cannot reconstruct signals, these two losses were not included in the comparison. Table 10 illustrates the comparison results for all reconstructions and datasets. We have also used a paired *t*-test statistical technique to detect whether the TSF-MSE based GAN/WGAN architectures significantly outperform than the temporal-MSE based GAN/WGAN architectures. In Table 10, *AO*−>*AO* means AO dataset reconstructed by the same sensitivity AO dataset, *GAL*−>*AO*/*MI*−>*AO* represents AO dataset reconstructed by the different sensitivity GAL/MI datasets, and so forth. Experimental results have shown that no matter GAN architecture or WGAN architecture, TSF-MSE loss function outperformed the conventional temporal-MSE loss function (*p <0.05). Therefore, the novel loss function proposed by us will significantly improve the performance of the reconstructed EEG signals.

**Table 10:** The comparison results between Temporal-MSE and TSF-MSE of constructing GAN/WGAN architectures for reconstruction.

**Reconstruction**	**Temporal-GAN (a)**	**TSF-GAN (b)**	**Temporal-WGAN (c)**	**TSF-WGAN (d)**
*AO*−>*AO*	64.86	65.41	64.32	**67.67**
*GAL*−>*GAL*	71.43	**74.64**	72.68	73.89
*MI*−>*MI*	62.43	63.61	63.83	**64.01**
*GAL*−>*AO*	64.16	**64.55**	63.85	64.40
*MI*−>*AO*	**62.31**	62.07	61.84	62.08
*AO*−>*GAL*	68.84	**70.60**	68.73	70.34
*MI*−>*GAL*	68.93	**70.21**	69.29	69.57
*AO*−>*MI*	62.35	**64.93**	62.86	63.29
*GAL*−>*MI*	62.19	63.39	62.28	**63.66**
*T*-test	–	b vs. a	–	d vs. c
*p*-value	–	**p <0.01	–	*p <0.05

*The bold text is the best performance*.

Classification of reconstructed signals between the “FBCSP+SVM” classifier and “FBCSPNet” classifier Schirrmeister et al. (2017) are illustrated in Table 11. The results have shown average classification results of “FBCSP+SVM” and “FBCSPNet” for both GAN and WGAN models on three datasets. The improved ratios have shown that the GAN model and WGAN model bring 3.75 and 5.25% improvement on the average, respectively, to all three datasets for the “FBCSP+SVM” classifier. In addition, the GAN model and WGAN model bring 1.68 and 2.21% improvement on average, respectively, for all three datasets for “FBCSPNet” classifier. Therefore, we have concluded that EEG signals reconstructions by GAN/WGAN model are advantageous to the classification performance for different classifiers. If the classifier exhibits the a better performance, it has the ability to obtain more discriminant ERD patterns, so the improvement of the deep learning classifier is less than the conventional classifier.

**Table 11:** The comparison average results of three datasets between FBCSP+SVM classifier and FBCSPNet classifier.

	**FBCSP+SVM**			**FBCSPNet**		
**Datasets**	**Original data**	**TSF-GAN**	**TSF-WGAN**	**Original data**	**TSF-GAN**	**TSF-WGAN**
AO dataset	63.57	65.41	67.67	67.29	68.41	68.84
GAL dataset	69.78	73.63	73.89	73.61	74.82	75.23
MI dataset	61.98	63.61	64.01	65.47	66.64	66.86
Average	65.11	67.55	68.52	68.79	69.95	70.31
Improved Ratio	–	3.75%	5.25%	–	1.68%	2.21%

**Figure 11:**
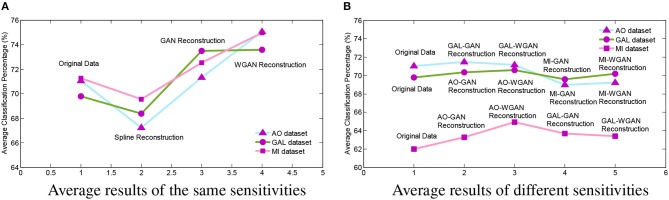
Reconstruction results comparison between the same and different sensitivity GAN/WGAN frameworks. In order to intuitively represent the differences between EEG signals reconstruction by the same sensitivities or different sensitivities of EEG signals, the average results of such compared experiments are illustrated to show the disciplines of EEG signals reconstruction by the GAN/WGAN models analyzed above. **(A)** Average results of the same sensitivities, **(B)** Average results of different sensitivities.

In order to intuitively represent the differences between EEG signals reconstruction by the same sensitivities or different sensitivities of EEG signals, Figure 11 illustrates the average results of these comparisons. In Figure 11, the Figure 11A shows the average results of Tables 4–6 and Figure 11B shows the average results of Tables 7–9. From the average figures, the disciplines of EEG signals reconstruction by the GAN/WGAN models analyzed above can be found.

## 4. Discussion

### 4.1. Reconstruction by Using GAN/WGAN Frameworks and TFS-MSE Loss

The purpose of this paper is to reconstruct HSS-EEG signals from LSS-EEG signals by using GAN/WGAN frameworks with a carefully designed loss function. In this paper, among the experiments of three different EEG datasets, we have compared the performance of GAN/WGAN frameworks for up-sampling with the same sensitivity and reconstruction with different sensitivities. The classification performances show significant improvement in terms of reconstructions of the same sensitivity. For AO dataset, the classification performances also show significant improvement by reconstructions of GAL-WGAN and GAL-GAN. However, other datasets reconstruction signals with different sensitivity have no significant improvement than original signals. There are two possible reasons for the statistical results. One possible reason is that the AO dataset has enough subsets (a total of 24) to compute the *t*-test index. However, datasets GAL and MI with 12 subsets and nine subsets, respectively, are not sufficient to compute the *t*-test index. Another possible reason may be due to the signal amplitude range for the GAN/WGAN reconstruction. In our experiments, the reconstructed signals amplitude range was set as the same as the original signals, and the amplitude range may have prevented the variations of reconstructed signals brought by the signals with different sensitivity. Therefore, in future works, more experiments for different ranges are also needed for a same dataset to confirm the relationship between signal amplitude range and patterns classification performance. For the average classification accuracy for all experiments, the up-sampled EEG signals performed better than the original data, and we think this might be due to the fact that the reconstruction procedure obtains more discriminant signals. In addition, the original temporal-MSE and the proposed TSF-MSE as loss functions were also compared.

The up-sampling reconstruction with the same sensitivity results have demonstrated that using the WGAN helps to improve signal qualities and statistical properties. Comparing the reconstruction HSS-EEG signals and the original real HSS-EEG signals in Figures 6, 8A, 9A, 10A, we can see that the WGAN framework helps to solve the problem of the over-smoothing effect suffered by the conventional temporal-MSE signal generators (Aydin et al., 2015). Although the reconstructed HSS-EEG signals shared a similar result, as in Figures 6, 8A, 9A, and 10A,B, the quantitative analysis of classifying signals by a machine learning model, as given in Tables 4–6, Figure 11A, have shown that the WGAN framework yields a higher classification accuracy and obtains more reliable statistical properties due to more discriminant patterns. However, if we use GAN/WGAN frameworks alone, the critical ERD/ERS of brain activity characteristics in the EEG signals will be reduced along with the single temporal loss. Theoretically, the GAN/WGAN frameworks are based on generative models, and such models generate naturally appearing HSS-EEG signals but cause severe distortion of the ERD/ERS characteristics in the EEG signals (Choi et al., 2017). Therefore, an additive loss function should be included to guarantee that the ERD/ERS characteristics remain the same for the reconstruction.

Beyond the above analysis, the TSF-MSE loss function was introduced to guarantee the ERD/ERS characteristics during the training of the GAN/WGAN frameworks, and the classification performance of ERD/ERS characteristics can be found in the compared results in Table 10. As is well known, the temporal-MSE loss was the basis of the time-series data, and such loss will guarantee the reconstructed shape of the temporal domain. However, EEG signals are multi-channel time-series data, and the spatial domain is thus also important in the reconstruction. In addition, most ERD/ERS characteristics are reflected in the frequency domain, making the frequency domain also important in the reconstruction. Therefore, the TSF-MSE constructed by the original signals from the temporal domain, the FB-CSP features from the spatial domain, and the PSD features from the frequency domain have been introduced in this paper to guarantee the EEG signals temporal characteristics, spatial characteristics, and ERD/ERS characteristics (Strohmeier et al., 2016). Additionally, the TSF-MSE-based GAN/WGAN models cause lower losses than the temporal MSE, frequency MSE, and spatial MSE-based GAN/WGAN models (see Figure 4). Our proposed TSF-MSE-based WGAN framework outperformed the other models in reconstructing up-sampled EEG signals with the same sensitivity. These results demonstrate that we can use this method to increase the sampling rate of EEG signals to achieve higher performance in brain-computer interfaces (BCIs) or EEG-based rehabilitation treatments.

### 4.2. EEG Signal Reconstruction With Different Sensitivities

In this paper, in addition to reconstructing HSS-EEG signals from the same sensitivity, we also reconstructed HSS-EEG signals from different sensitivities. In fact, if EEG signals with low sensitivity can be reconstructed into high-sensitivity signals, the reconstructed HSS-EEG signals will contain more details of the ERD/ERS characteristics, which will improve the classification performance for many applications. Among the experimental results shown in Tables 4–9, we can conclude that the average classification accuracies of WGAN framework are higher than GAN framework for reconstruction with the same sensitivity on all datasets, while the GAN framework obtained better average classification accuracies for reconstruction with different sensitivities on all datasets. In addition, a larger gap in the sensitivity will significantly increase the average classification accuracies of all datasets, while a smaller gap in the sensitivity will result in a smaller difference in the average classification accuracies of all datasets (see the comparison results in Tables 7–9, Figure 11B). We can also find indicators for different sensitivity gaps in Figure 7. For example, considering the AO data reconstructed by the MI-GAN and MI-WGAN models (see Figure 7B), a high-sensitivity signal reconstructed by the low-sensitivity GAN/WGAN models caused the signals to be overfitted and exceed the original data range. Hence, the reconstructed results contained fewer ERD/ERS characteristics to classify the EEG signals, and the classification accuracy was lower than the results using the original data. Conversely, for the MI data reconstructed by the AO-GAN and AO-WGAN models (see Figure 7F), we can see that the low-sensitivity MI data reconstructed by the high sensitivity models presented more variations in the temporal domain. Because the variations in the time-series represented detailed characteristics of ERD/ERS, the reconstructed high sensitivity EEG signals performed better in the classification of ERD/ERS characteristics. Therefore, in practical applications, we can train a high-sensitivity GAN model for EEG signal reconstruction. By applying the GAN/WGAN models, the ERD/ERS characteristics extracted from low sensitivity devices can be enhanced for use in real-time and real-application BCI or rehabilitation treatment.

In contrast to the results of reconstructing HSS-EEG signals with the same sensitivity, the GAN framework performed better than the WGAN framework for reconstructing HSS-EEG signals with different sensitivities. An approaching value range caused a smaller difference between the GAN framework and the WGAN framework (the AO dataset and the GAL dataset), but a separated value range caused a large difference between the GAN framework and the WGAN framework. Therefore, the difference in the classification performance was caused by the different value ranges of different sensitivities. We suggest two reasons for this difference: first, the WGAN framework contained a gradient penalty, and such a penalty would be out of the value ranges for different value ranges. The penalty then influenced the convergence of the WGAN framework (Mescheder et al., 2018), and, thus, the results of the WGAN framework were lower than the results of the GAN framework. Second, the WGAN framework used an RMSprop optimizer to train deep neural networks, but the GAN framework used an Adam optimizer (Basu et al., 2018). In fact, the Adam optimizer has a momentum gradient procedure, which will be fitted for regressing different value ranges. Hence, the different value ranges can be reconstructed by the Adam optimizer (Zou et al., 2018). In all of these, if we have recorded the highest sensitivity EEG signals, we must also record low-sensitivity EEG signals. We can use the highest sensitivity EEG signals to train a GAN/WGAN model to reconstruct the low sensitivity EEG signals, and the reconstructed EEG signals can be used to improve classification performance for the construction of real-time and real-application BCIs or rehabilitation treatment.

### 4.3. The Application of Reconstructed EEG Signals by GAN/WGAN Frameworks

Over the past decade, most EEG-based studies have been focused on constructing BCIs or developing rehabilitation treatments (Ang et al., 2015). However, there are two main limitations to the application of EEG signals when constructing such systems, namely, the cost and portability of EEG recording devices. In fact, HSS-EEG signals will yield the best performance in BCIs and rehabilitation treatments, although HSS-EEG signals are usually recorded by expensive devices, posing an inconvenience. For example, in the “NeuroScan SymAmp2” device (Chu et al., 2016), the recording system consists of two computers and one device to link them together. One computer is used to present a stimulus for the BCI or rehabilitation treatment, and the other computer is used to record and store the EEG signals for computing the BCI or rehabilitation results. Subjects must sit in a room to wear a “NeuroScan Quik Cap” to collect data. The collection procedure is complex, and the resistance must be maintained under 5 kΩ by using conductive paste on each electrode (Agnew et al., 2012). Because the resistance is kept low and the device has a high sensitivity, the recorded EEG signals will have the ERD/ERS characteristics required for classification in BCI and rehabilitation treatment.

In general, the “NeuroScan SymAmp2” device is expensive, and the EEG signals must be recorded indoors in a limited environment (e.g., a dimly lit, sound-attenuated room). Hence, it is difficult to implement the results of the “NeuroScan SymAmp2” device (the same sensitivity as signals in AO dataset) in applications such as BCI and rehabilitation treatment. Nevertheless, low-cost and portable devices, such as “Emotiv” (the same sensitivity as signals in MI dataset), have high electrode resistance and a low sampling rate and sensitivity for recording EEG signals. The device only provides poor ERD/ERS characteristics for classification in BCI and rehabilitation treatment applications. The “Emotiv” device can be worn at any time via a simple process without requiring the resistance to be kept level (Neale et al., 2017). The energy supply for the device is a battery, and the device uses WiFi or Bluetooth communication. These advantages allow the device to be inexpensive, portable, and convenient for constructing BCIs and developing rehabilitation treatment. These mutual contradictions for signal precision and signal cost and portability inspire us to train a model to reconstruct HSS-EEG signals from LSS-EEG signals. The trained model meets the requirements of high precision and portability with low cost and can be used to improve EEG-based applications.

In fact, signal reconstruction is a difficult problem in digital signal processing, but an effective and feasible reconstruction method could significantly promote the application of signals. In this study, by using a GAN framework with Wasserstein distance and the carefully designed TSF-MSE loss function, well-trained reconstruction models have been shown to be able to reconstruct HSS-EEG signals from LSS-EEG signals. Experimental results reveal that LSS-EEG signals (just like those recorded by “Emotiv”) reconstructed by the HSS-EEG signals (just like those recorded by “NeuroScan SymAmp2”) trained models and enhanced the average classification accuracies of ERD/ERS characteristics for action observation, action execution, and motor imagery. These results inspire new ways to construct BCIs or develop novel rehabilitation treatments, but more researches need to be done to explore significant enhancement reconstruction methods across EEG signals with different sensitivities.

Based on the method of this paper, the improvement of sampling rate and sensitivity will improve the specific ERD/ERS phenomenon of MI, AO, and AE, so as to improve the performance of the BCI system. Although the CNN- based GAN/WGAN architectures will take a significant amount of time to build an available GAN/WGAN architecture, once the reconstruction model is built, the use of such a model will not take long, and the reconstructed EEG time series can be obtained within a specific time (<1 s for a trial). In future works, we can either reduce the complex of GAN architecture or improve the computational efficiency to reduce the usage time for reconstructing GAN/WGAN architecture. Then, the GAN/WGAN architectures will be used for real-time inference. In general, we used a low-cost, portable device to collect LSS-EEG signals for use in BCI or rehabilitation treatment. Before analyzing the collected data, the GAN/WGAN reconstruction models were applied to reconstruct HSS-EEG signals. The reconstructed HSS-EEG signals can significantly improve the classification performance and information transfer rate for use in BCIs or rehabilitation treatments.

## 5. Conclusion

In this paper, we have proposed a contemporary deep neural network that uses a GAN/WGAN framework with a TSF-MSE-based loss function for LSS-EEG signal reconstruction. Instead of designing a complex GAN framework, this work has been dedicated to designing a precise loss function that guides the reconstruction process so that the reconstructed HSS-EEG signals are as close to the ground truth as possible. Our experimental results suggest that the GAN/WGAN frameworks give a significant improvement on the classification performance of EEG signals reconstruction with the same sensitivity, but the classification performance improvements of EEG signal reconstructions with different sensitivity were not significant, which further exploration. The carefully designed TSF-MSE-based loss function solves the well-known over-smoothing problem and seems to result in more discriminant patterns than the original EEG signals; this will improve the classification performance of EEG signals. The reconstructed HSS-EEG signals will be beneficial for use in BCI and rehabilitation treatment applications. Future studies will focus on the reconstruction signal amplitude ranges of EEG signals with different sensitivity and selection of datasets to confirm the required number of signals and to explore the significant performance improvement of EEG signal reconstruction with different sensitivity. In addition, the efficiency of EEG signal reconstruction by the GAN/WGAN frameworks will be studied further in the future.

## Data Availability Statement

The datasets generated for this study can be found in:

1. https://pan.baidu.com/s/1NC4-ywOssfX2nUMaEeGlRw please use the extract code “g8ip” in the dialog box to access the Datasets.
2. https://www.kaggle.com/c/grasp-and-lift-eeg-detection
3. BCI Competition IV “Dataset 2b”: http://www.bbci.de/competition/iv/#datasets.

## Author Contributions

TL and YF designed the experiments. YF and LC completed the experiments. TL and GG analyzed the EEG data. CZ, LC, GG, and TL wrote the paper.

## Conflict of Interest

The authors declare that the research was conducted in the absence of any commercial or financial relationships that could be construed as a potential conflict of interest.

## Footnotes

^1^https://pan.baidu.com/s/4gap5N4.

^2^https://www.kaggle.com/c/grasp-and-lift-eeg-detection/data.

^3^http://www.bbci.de/competition/iv/#datasets.

## Abbreviations

EEG: electroencephalography
HSS-EEG: high sampling rate and sensitivity EEG
LSS-EEG: low sam-pling rate and sensitivity EEG
DNNs: deep neural networks
GAN: generative adversarial network
WGAN: GAN with Wasserstein distance
HR: high resolution
LR: low resolution
FBCSP: filter bank common spatial pattern
PSD: power spectral density
TSF-MSE: temporal-spatial-frequency mean square error
AO: action observation
GAL: grasp and lift
MI: motor imagery
ReLU: rectified linear unit
BN: batch normalization
SVM: support vector machine
SVD: singular value decomposition
ERD/ERS: event-related desynchronization/event-related synchronization.
